# Postinactivity Exercise Training Improves Sarcopenia Traits in 40–60‐Year‐Old Women Regardless of Fortified Milk Supplementation

**DOI:** 10.1002/jcsm.70080

**Published:** 2025-11-03

**Authors:** Joanne Trezise, Ricardo M. Lima, Sally D. Poppitt, Aaron C. Fanning, Amanda Devine, Anthony J. Blazevich

**Affiliations:** ^1^ Human Performance Centre Southern Institute of Technology, Te Pūkenga Southland New Zealand; ^2^ Department of Nutrition University of Brasília Brasília Brazil; ^3^ Department of Physical Education University of Brasília Brasília Brazil; ^4^ Human Nutrition Unit, School of Biological Sciences The University of Auckland Auckland New Zealand; ^5^ Fonterra Research and Development Centre Palmerston North New Zealand; ^6^ Nutrition and Health Innovation Research Institute, School of Medical and Health Sciences Joondalup WA Australia; ^7^ Centre for Precision Health, School of Medical and Health Sciences Edith Cowan University Joondalup WA Australia

**Keywords:** disuse, fortified milk product, menopause, physical function, sarcopenia, step count

## Abstract

**Background:**

The term sarcopenia was introduced to describe the age‐related decline in muscle mass, but current definitions also include measures of muscle strength and function. Menopause increases sarcopenia risk and may exacerbate the adverse effects of physical inactivity. Exercise training is a potent stimulus to restore muscle health, and nutritional supplementation can further improve the outcomes. The purposes of this study were to examine the changes in a set of sarcopenia‐related phenotypes induced by a 14‐day step‐reduction period followed by a 12‐week exercise training programme, with or without fortified milk supplementation in healthy 40–60‐year‐old females, and to determine whether menopausal status interacted with these changes.

**Methods:**

In this double‐blind, placebo‐controlled randomized trial, females aged 40–60 years were evaluated before and after 2 weeks of reduced activity monitored through pedometer and after a subsequent 12‐week exercise + nutrition programme with ingestion of a fortified milk product (FMP) or placebo. Muscle volume (dual‐energy X‐ray absorptiometry [DXA] and peripheral quantitative computed tomography), handgrip (hydraulic handheld dynamometer), knee extensor and plantar flexor strength (isokinetic dynamometer) and a variety of physical function measures were assessed at all timepoints.

**Results:**

Eighty‐three self‐reported healthy females (50.7 ± 5.3 years; 52 postmenopausal) completed the reduced‐activity period, and 67 completed the subsequent exercise training + nutrition phase. At baseline, participants averaged 8323 ± 3077 daily steps and then decreased to 1876 ± 729 during the reduced‐activity period. Mean sarcopenia outcomes declined after 2 weeks of activity restriction, with significant changes (*p* < 0.05) in shank muscle cross‐sectional area (CSA) (67.7 ± 9.9 to 66.5 ± 9.9 cm^2^), handgrip strength (25.3 ± 5.0 to 24.0 ± 5.3 kg), knee extensor peak torque (134.7 ± 36.2 to 122.7 ± 34.5 Nm) and stair ascending time (3.6 ± 0.6 to 3.7 ± 0.6 s) and power (367.6 ± 67.6 to 356.7 ± 67.7 W), with no significant time × menopause interactions (*p* > 0.05 for all variables). All muscle mass, strength and function outcomes were not only improved after exercise training + nutrition but also significantly increased compared to preintervention baseline (all *p* < 0.05). No training × menopause or training × supplementation interactions were observed for any variable (both *p* > 0.05).

**Conclusions:**

Two weeks of step reduction negatively affected muscle mass, strength and physical function in 40–60‐year‐old females. A 12‐week training programme including strength exercises and dietary supplementation not only recovered muscular health but also promoted improvements above the baseline before the physical activity restriction. Menopause status did not influence changes in response to step reduction or exercise, and the addition of a fortified milk product during the training period did not influence the induced adaptations.

## 1 Introduction

Skeletal muscle health is vital for the maintenance of physical function and independence with advancing age. The term sarcopenia was introduced in 1989 to describe the age‐related decline in skeletal muscle mass [1], and the condition received its own International Classification of Disease code in 2016 (M62.84) [2]. Sarcopenia is associated with numerous adverse clinical outcomes [3, 4, 5, 6, 7], imposing an important economic burden on health care costs [8, 9, 10]. However, a consensual operational diagnosis has yet to emerge. Several expert committees [1, 4, 11, 12, 13, 14] have been created to fill this gap, but a global consensus is still lacking. Because muscle strength and function are more strongly associated with clinical outcomes than muscle mass, these committees agree that measures of muscle strength and physical function should be incorporated into the sarcopenia operational diagnosis [1, 4, 11, 12, 13, 14]. Given that females have less muscle mass and strength than men over the lifespan and undergo relatively abrupt hormonal changes with menopause, they may be more prone to develop sarcopenia [15, 16].

Sarcopenia has long been considered a geriatric syndrome; however, its trajectory commences earlier in life [1, 11, 15] and both earlier and more rapidly in females [15, 17], partially as a result of the menopause transition. Menopause‐related changes in hormonal status, particularly oestrogen level reduction, negatively affect muscle mass and strength [15, 17]. Nonetheless, increased physical activity is associated with better muscular health in pre‐ and postmenopausal middle‐aged females, supporting the proposition that being physically active during midlife may decrease the sarcopenia risk with advancing age [15, 18]. However, episodic periods of reduced physical activity due to work travel, physical injury or hospitalization, caring responsibilities or inclement weather may further contribute to atrophy and potentially negatively impact an individual's muscle strength and function. Reidy et al. [19] observed leg lean mass, midplantar flexor muscle area and quadriceps strength were all significantly decreased after 14 days of reduced daily physical activity (step counts in this case) in healthy 60–85‐year‐olds. Conversely, 2 weeks of step reduction was not detrimental to muscle strength and function in healthy adults with a mean age of 72.5 years [20]. Yet another study [21] found that older men (67.3 years) showed an attenuated rate of muscle atrophy in response to immobilization but also an impaired ability to restore muscle size and strength over a subsequent retraining period compared to young men (24.4 years). These findings have since been corroborated by other groups [22, 23]. With specific reference to females, results from animal models suggest that ovarian function affects the recovery of skeletal muscle after a period of reduced physical activity [24], but clinical data in humans are scarce. The effects of an episodic period of physical activity reduction on the progression of sarcopenia in middle‐aged females thus remain unclear.

Because menopause transition can exacerbate the adverse effects of disuse on muscle morphology and function, countermeasures to mitigate muscle mass, strength and function loss are particularly relevant. Exercise training, particularly involving strength training, is a potent stimulus to restore muscle health, but optimizing nutritional intake via protein ingestion, food fortification and nutritional supplementation may further improve the outcomes. For example, a synergistic effect of milk (alone or fortified) ingestion and exercise training has been found on net muscle protein synthesis [25, 26], although data are not consensual [27, 28]. The processing of milk allows for the development of fortified products with higher nutrient levels, but no previous studies have investigated its effects in restoring muscle function in females around menopause.

Given the above, the purpose of this study was to examine changes in a comprehensive set of sarcopenia‐related phenotypes induced by a 14‐day step‐reduction period followed by a 12‐week exercise training programme with fortified milk product (FMP) supplementation or isoenergetic placebo in middle‐aged females. As a secondary aim, we sought to determine whether menopausal status would interact with these changes.

## 2 Methods

A detailed description of the methods is provided in Supporting Information, including participant recruitment, sample size calculations, full exclusion criteria, assessment procedures and measurement protocols. Key elements are summarized below to ensure clarity in the main text.

### 2.1 Study Overview

After completing a comprehensive familiarization session in advance of the first testing session, participants were fully evaluated at three timepoints: before physical activity reduction (T1), after 2 weeks of physical activity reduction (T2) and after a subsequent 12‐week exercise training programme (T3) with or without supplementation of either the FMP or the placebo. During the 14‐day reduced physical activity period, participants were encouraged to reduce their physical activity to below 1500 steps per day and had steps recorded each day using a pedometer to assess compliance. No participants performed other deliberate exercise activities. Immediately after this period, participants were randomized into a 12‐week exercise training programme + one of the supplements, in a double‐blind fashion (see Supporting Information for details). The project protocol was registered with the Australia and New Zealand Clinical Trial Register (ACTRN12616001714471).

### 2.2 Participants

Participants aged 40 to 60 years were recruited via community flyers, local radio and newspaper advertisements and direct email. A total of 528 females answered the study call from which 156 were eligible and invited to attend an informative session for details. Exclusion criteria included body mass index (BMI) < 18 or > 35 kg/m^2^, being engaged in regular exercise training, a previous negative reaction to milk or dairy proteins and currently performing structured physical activity in their occupation; full criteria are listed in Supporting Information. After exclusion criteria were applied, 93 volunteers were enrolled in the study, from whom 83 completed all the assessments performed immediately after the reduced step phase. Volunteers were randomized to supplement‐plus‐exercise or placebo‐plus‐exercise groups for 12 weeks, with 67 participants completing the intervention and all subsequent assessments.

**FIGURE 1:**
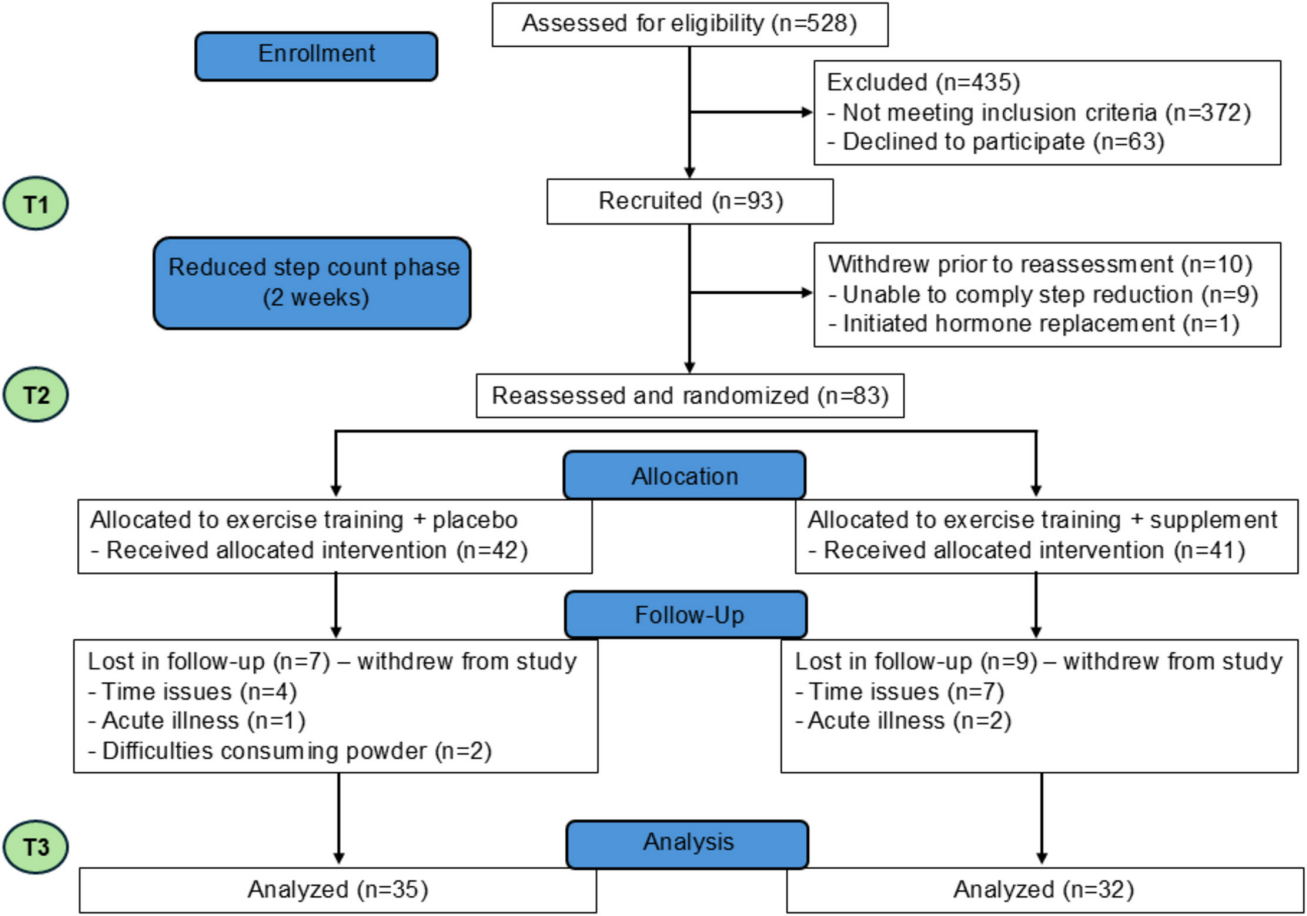
Flow diagram for participant recruitment and assignment in the present study.

Reasons for study withdrawal included difficulties complying with the reduced activity period, initiation of hormone replacement therapy, acute illness, injuries unrelated to the study, difficulties consuming the supplement and personal or family reasons, as detailed in the participant flow diagram in Figure 1. All volunteers answered a questionnaire regarding their medical history and lifestyle habits. For menopausal status, ‘postmenopausal’ was defined as at least 12 months without having a period. All participants were informed of the study procedures in person and voluntarily signed an informed consent form. The experiments were conducted in accordance with the Declaration of Helsinki, and the study protocol was approved by the Edith Cowan University Review Board (13317).

To determine habitual physical activity and dietary habits, baseline measurements were taken before the reduced activity phase using a 3‐day habitual activity record, including wearing a pedometer (G Sensor Pedometer, H‐216G MVPA, Paris, France) on the waist secured by an elastic belt during waking hours to record daily step counts. Participants also completed a 3‐day weighed dietary record. Participants then returned to the laboratory for two consecutive testing days to complete body composition, strength and functional measurements.

### 2.3 Reduced Step Period

Participants were instructed to reduce their physical activity to below 1500 steps per day over 2 weeks and spend most of their day engaged in sedentary behaviour. It was recommended to spend less than 90 min/day engaged in light activities, such as slow walking, cooking food and washing dishes, and to refrain from all moderate‐ and high‐intensity activity. Daily step counts were evaluated using the pedometer throughout the 2 weeks, with pedometers checked daily by walking 20 steps and then checking pedometer count accuracy.

### 2.4 Exercise Training Programme

Immediately after the reduced step period, the requirement to restrict daily steps was removed and participants began a 12‐week exercise training programme in combination with fortified milk or placebo powder supplementation, in addition to participants' habitual daily physical activity. The training programme included four training sessions per week, with two university‐based, supervised sessions completed in small groups of up to eight participants and two home‐based, self‐supervised sessions performed individually. The protocol included elements of traditional strength and flexibility training plus general fitness exercise. For university‐based sessions, participants completed 50 min of broad‐based exercise training including muscle stretching, balance, muscle strength and physical endurance exercises. Sessions commenced with mobility and muscle stretching exercises (15 min). Fitness and muscle strength training followed, and the session was concluded with a short (5 min) stretch and warm‐down period. For the fitness/endurance component (8 min per session), participants performed stair climbing, cycling, rowing and/or walking/running at a ‘moderate intensity’ in intervals with a 1:3 high‐ vs. low‐intensity work ratio (e.g., cycle 15 s at Level 12 and then 45 s at Level 4). The exercises were individualized and progressed in intensity across the 12 weeks. Strength training (20 min) was implemented using both traditional machines (e.g., chest, back and leg press) and the use of dumbbells (e.g., standing shoulder press, squats and lunges), focused on major muscle groups and was completed in a circuit format with 45 s:15 s work:rest. Within each 45 s, 10–15 repetitions were performed at an intensity of 14–15/20 RPE. All trainers held at least a degree‐level qualification in Exercise and Sport Science and completed an attendance log for all training sessions. At home, participants performed a 20–25 min session that mirrored the movements and exercises performed in the university gymnasium, but which could be achieved without supervision or equipment. A reference sheet with photographic explanations was supplied as reference. Home‐based sessions began with a 10‐min warm‐up of active range of motion exercises and then performed ~10 min body mass‐resistance exercises including single sets of squats, lunges, push‐ups and supermen (lifting arms and legs into extension while lying prone on the floor) until reaching a 17–20 rating of exertion (6–20 scale), then 1 min of cobra (push up to arch back while lying prone) and donkey‐kick (single‐leg extensions from hip while on all‐fours) exercises. Participants were also instructed to complete at least 3000 steps per day on nonexercise days to encourage maintenance of their habitual physical activity and to reinforce that the training programme was supplementary to their usual routine.

### 2.5 Muscle Strength Evaluation

Muscle strength was evaluated bilaterally using handgrip strength (HGS; Jamar hydraulic dynamometer; Sammons Preston Rolyan, IL, USA) and both knee extensor and plantar flexor isometric torques (isokinetic dynamometer; Biodex 4, Biodex Medical Inc., NY, USA). The mean of the highest value of each arm in kilograms (HGS) and absolute and normalized (to body mass) isometric torques at each joint was recorded. The test–retest coefficients of variation (CV) for handgrip (HGS), knee extension and plantarflexion were 2.9%, 2.1% and 5.1%, respectively. Details regarding muscle strength evaluations are presented in Supporting Information.

### 2.6 Physical Function Tests

The timed up and go (TUG; CV = 3.0%), 6‐m walk tests (6‐MWT; CV = 3.1%), 11‐stair ascent (CV = 3.4%) and squat jump (CV = 2.5%) tests were performed. Considering that participants in the present study were middle‐aged, physical performance evaluations additional to those widely used in older cohorts were conducted, including squat jump height and stair climb performance. Assessment details are reported in Supporting Information.

### 2.7 Peripheral Quantitative Computed Tomography

Lower leg (shank) and thigh whole‐muscle CSAs were measured using peripheral quantitative computed tomography (pqCT; XCT 3000; Stratec Medizintechnik, Pforzheim, Germany). Thigh muscle CSA was measured at the midpoint between the lateral epicondyle and greater trochanter, while shank CSA was scanned at levels of 4% and 66% from the distal end of the tibia. Due to equipment technical difficulties during the study, pqCT evaluations for thigh and shank were successfully completed in 36 and 46 participants, respectively, at all timepoints. Scanner performance was assessed through daily phantom scans, which resulted in a coefficient of variation of 0.14% for total attenuation across repeated measurements. Test–retest coefficient of variance is 1.3% for muscle CSA in our laboratory.

### 2.8 Body Composition Assessment

Body mass and height were measured using standard procedures and BMI was calculated. Body composition was measured using dual‐energy X‐ray absorptiometry (DXA; Horizon A, Hologic, Washington, USA). Appendicular lean mass was calculated as the sum of both arms and legs. Lean body mass (LBM) was also calculated relative to body mass (%), while appendicular lean mass was calculated relative to height squared (kg/m^2^). Percent body fat characterized each participant's adiposity. The equipment was calibrated daily according to manufacturer specifications, and all procedures were conducted by the same trained professional. Coefficients of variation for LBM and fat mass are 0.3% and 0.9%, respectively, in our laboratory.

### 2.9 Supplementation

Participants were randomly assigned to a FMP or placebo supplementation group. Both supplements were ingested in two 30‐g doses per day mixed with 150‐mL water. The FMP was largely comprised milk powder (skim and whole) and contained 9‐g protein, 16.5‐g carbohydrate, 0.7‐g fat, 634‐mg calcium, 7.5‐μg vitamin D and extra vitamins and minerals (see Supporting Information). The placebo was designed to balance the requirements for mass and textural as well as energy content similarity to the supplement powder (500.3 vs. 459.2 kJ per dose, respectively) and predominantly contained rice and whole milk powders with 0.95‐g protein, 26.5‐g carbohydrate, 0.9‐g fat, 29‐mg calcium and no added vitamins or minerals. The supplement was ingested additional to meals, not as a replacement, with breakfast and lunch on nonexercise days but with one dose within 30 min of exercise completion on their exercise days. Participants were required to report any exercise‐ or supplement‐related adverse events. Adherence was determined by counting returned powder packets and weighing residual power and calculated as a percentage consumed relative to amount prescribed.

### 2.10 Diet Records

Three‐day weighed dietary records were obtained across two weekdays and one weekend day using a booklet with pages for recording each day's food intake at T1, during the final 3 days of reduced activity (pre T2) and during the 11th week of training (pre T3). All food and beverage details were recorded, including a description of the foods, brand names and food mass or amounts consumed (in grams, cups, teaspoons, pieces, slices, etc.). Data were analysed using the Foodworks 8 software (Xyris Software, Australia).

### 2.11 Sample Size

Sample size estimation was based on ascending stair climb time due to its functional importance and requirement for muscle size/strength. Research papers relating to changes in strength and power performance revealed generally large effect sizes ranging from 1.2 to 1.6. Using a conservative effect size of 0.8 (due to our younger population, lower protein in the supplement and training less focused on heavy strength training), the total sample size required was 68, as described in detail in the Supporting Information. Considering a possible dropout of 20%, 82 participants were required to begin the study.

### 2.12 Statistical Procedures

Participant characteristics are presented as means and standard deviations, unless otherwise noted. To examine the effects of the reduced step period (T1 to T2) on outcome variables, linear mixed models (LMMs) were used, with participant identification as a random factor, then timepoint, menopause and age as fixed factors and each outcome variable as a dependent variable. LMMs were also used to examine the effects of exercise training (T2 to T3) with FMP or Placebo on dependent variables, in which group (i.e., supplement vs. placebo) was entered as a fixed factor. Analyses were repeated including menopausal status and age as fixed factors. To address the potential influence of noncompliance with the 1500 daily step count target on the outcomes, an additional LMM was performed in which compliance (yes or no) was entered as a fixed factor along with time, while dependent variables were retained. Comparisons of continuous variables at baseline between pre‐ and postmenopausal participants were performed using general linear models, which were repeated using age as a covariate. Coefficients of variation were calculated using T1 data. Data were considered significant at *p* < 0.05, and statistical analyses were performed using SPSS software version 29.0.

## 3 Results

Participants' baseline characteristics for the entire sample and according to menopausal status are presented in Table 1. Eighty‐three middle‐aged participants successfully completed the reduced step phase and assessments (T1 and T2), from which 31 were premenopausal and 52 postmenopausal. At T1, postmenopausal volunteers had significantly higher age and percent body fat but did not differ in body mass, height or BMI. Mean LBM relative to body mass, thigh muscle CSA (with trend for shank CSA; *p* = 0.07), HGS, HGS relative to body mass, knee extensor and plantar flexor isometric torques, squat jump height and ascending stair time and power were better for the pre‐ than postmenopausal group. When age was entered as a covariate, significant differences were still noted for usual gait speed, stair ascending time and stair ascending power (*p* < 0.05).

**TABLE 1:** Participant baseline sarcopenia trait characteristics for the entire sample and according to menopausal status (*N* = 83). Values are expressed as mean ± standard deviation.

Variable	Entire sample	Premenopausal	Postmenopausal
*N* (%)	83 (100)	31 (37.4)	52 (62.6)
Age (years)	50.7 ± 5.3	45.6 ± 3.5	53.8 ± 3.5*
Body mass (kg)	74.7 ± 13.3	73.5 ± 14.8	75.2 ± 12.6
Height (m)	1.64 ± 0.1	1.64 ± 0.1	1.64 ± 0.1
BMI (kg/m^2^)	27.7 ± 4.6	27.2 ± 4.9	28.0 ± 4.4
Percent body fat (%)	37.6 ± 5.4	36.1 ± 5.4	38.4 ± 5.2*
Muscle mass variables			
LBM (kg)	44.2 ± 5.7	44.5 ± 6.3	44.0 ± 5.3
LBM/body mass (%)	59.8 ± 5.2	61.3 ± 5.3	58.9 ± 5.0*
Appendicular LBM (kg)	18.7 ± 2.8	18.8 ± 3.0	18.6 ± 2.7
Appendicular LBM/height^2^ (kg/m^2^)	6.9 ± 0.9	7.0 ± 0.9	6.9 ± 0.9
Thigh muscle CSA (cm^2^)^a^	104.7 ± 15.7	108.6 ± 15.2	102.9 ± 16.1*
Thigh muscle density (HU)^a^	76.6 ± 1.8	76.9 ± 1.5	76.4 ± 2.1
Shank muscle CSA (cm^2^)^b^	67.7 ± 9.9	69.8 ± 11.9	66.5 ± 9.2
Shank muscle density (HU)^b^	77.2 ± 1.4	77.1 ± 1.4	77.2 ± 1.4
Muscle strength variables			
HGS (kg)	25.3 ± 5.0	26.4 ± 4.6	24.5 ± 5.1*
HGS/body mass	0.35 ± 0.1	0.37 ± 0.1	0.33 ± 0.1*
Knee extensor PT (Nm)	134.7 ± 36.2	139.7 ± 43.4	129.4 ± 30.1*
Knee extensor PT/body mass (Nm/kg)	1.8 ± 0.5	2.0 ± 0.5	1.7 ± 0.5*
Plantar flexor PT (Nm)	88.7 ± 30.8	99.1 ± 32.9	81.7 ± 28.5*
Plantar flexor PT/body mass (Nm/kg)	1.2 ± 0.5	1.4 ± 0.6	1.1 ± 0.4
Physical function variables			
Usual gait speed (m/s)	1.4 ± 0.2	1.4 ± 0.2	1.3 ± 0.2
Squat jump height (cm)	14.1 ± 4.5	15.6 ± 3.5	13.2 ± 4.9*
Stair ascending time (s)	3.6 ± 0.6	3.3 ± 0.6	3.7 ± 0.5*
Stair ascending power (W)	367.6 ± 67.6	387.5 ± 79.2	355.9 ± 57.7*
TUG (s)	5.4 ± 0.6	5.3 ± 0.6	5.5 ± 0.6

*Note:* *Significantly different to baseline (*p* < 0.05). a, *N* = 36; b, *N* = 46 [Note: these refer to the sample sizes available for these specific tests, where *N* is the sample size, as described in Methods].

Abbreviations: CSA, cross‐sectional area; HGS, handgrip strength; LBM, lean body mass; PT, peak torque; TUG, timed up and go test.

* Denotes significantly different to premenopausal group (*p* < 0.05).

Before the reduced activity phase, participants performed an average of 8323 ± 3077 daily steps, which decreased (*p* < 0.01) to 1876 ± 729 over the 2‐week reduced activity period, representing an average decline of 77.5%. Twenty‐seven participants (32.5%) met the criterion of < 1500 steps per day, while 61 (73.5%) remained below 2000 steps per day. In general, mean sarcopenia‐related traits were lower after the reduced step period, with statistical significance noted for HGS, knee extensor PT and both stair ascending time and power (Table 2). Limited changes were observed for skeletal muscle size measures, with only shank CSA presenting a statistically significant decline (−2.9%; *p* < 0.01). LMM did not reveal significant time × menopause or time × age interactions. Compliance with the < 1500 daily step target also did not interact with time. Over the reduced step period, total energy intake was lower than baseline (7554.8 to 7003.8 kJ, *p* = 0.003), mainly driven by reduced carbohydrate (175.1 to 78.4 g/day, *p* = 0.031) and protein (84.2 to 78.4 g/day, *p* = 0.047) intakes.

**TABLE 2:** Participant sarcopenia traits before (T1) and after (T2) 2 weeks of reduced daily step count (*N* = 83). Values are expressed as mean ± standard deviation.

Variable			
	T1	T2	∆%
Muscle mass variables			
LBM (kg)	44.2 ± 5.7	44.1 ± 5.8	−0.2
LBM/body mass (%)	59.8 ± 5.2	59.7 ± 5.1	−0.2
Appendicular LBM (kg)	18.7 ± 2.8	18.6 ± 2.9	−0.6
Appendicular LBM/height^2^ (kg/m^2^)	6.9 ± 0.9	6.8 ± 0.9	−0.6
Thigh muscle CSA (cm^2^)^a^	104.7 ± 15.7	102.6 ± 14.6	−2.0
Thigh muscle density (HU)^a^	76.6 ± 1.8	76.7 ± 1.9	0.1
Shank muscle CSA (cm^2^)^b^	67.7 ± 9.9	66.5 ± 9.9*	−2.9
Shank muscle density (HU)^b^	77.2 ± 1.4	77.4 ± 1.2	0.3
Muscle strength variables			
HGS (kg)	25.3 ± 5.0	24.0 ± 5.3*	−5.1
HGS/body mass	0.35 ± 0.1	0.33 ± 0.1*	−5.7
Knee extensor PT (Nm)	134.7 ± 36.2	122.7 ± 34.5*	−8.9
Knee extensor PT/body mass (Nm/kg)	1.8 ± 0.5	1.7 ± 0.5*	−5.6
Plantar flexor PT (Nm)	88.7 ± 30.8	87.3 ± 30.5	−1.6
Plantar flexor PT/body mass (Nm/kg)	1.2 ± 0.5	1.2 ± 0.4	0.0
Physical function variables			
Usual gait speed (m/s)	1.4 ± 0.2	1.3 ± 0.2	−7.1
Squat jump height (cm)	14.1 ± 4.5	13.7 ± 3.7	−2.8
Stair ascending time (s)	3.6 ± 0.6	3.7 ± 0.6*	2.8
Stair ascending power (W)	367.6 ± 67.6	356.7 ± 67.7*	−3.0
TUG (s)	5.4 ± 0.6	5.5 ± 0.6	1.8

*Note:* *Significantly different in relation to baseline (*p* < 0.05). a, *N* = 36; b, *N* = 46.

Abbreviations: CSA, cross‐sectional area; HGS, handgrip strength; LBM, lean body mass; PT, peak torque; TUG, timed up and go test.

**FIGURE 2:**
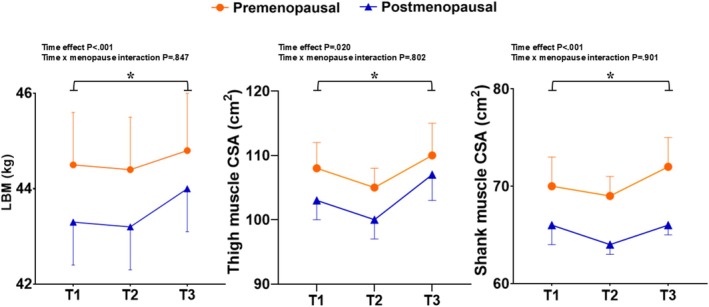
Muscle mass variables before (T1) and after (T2) 2 weeks of reduced physical activity and after a subsequent 12 weeks of exercise training (T3), according to menopausal status. (A) Whole body nonosseous lean body mass; (B) midthigh muscle cross‐sectional area; and (C) shank muscle cross‐sectional area. Circles (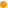) represent values for premenopausal participants and triangles (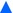) for postmenopausal. Values are mean ± SE. * Denotes that T3 is significantly different (*p* < 0.05) than T1.

**FIGURE 3:**
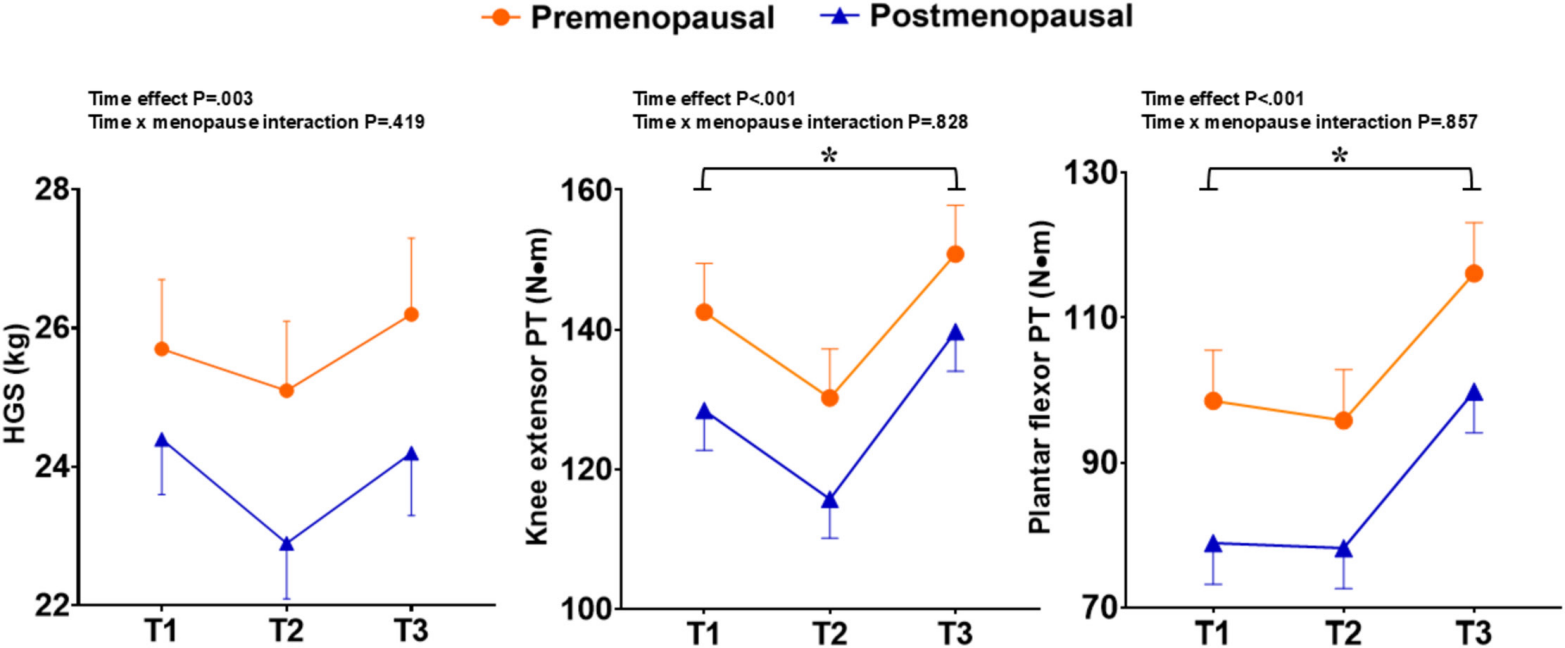
Muscle strength variables before (T1) and after (T2) 2 weeks of reduced physical activity and after a subsequent 12 weeks of exercise training (T3), according to menopausal status. (A) Handgrip strength; (B) knee extensor isometric peak torque; and (C) plantar flexor isometric peak torque. Circles (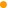) represent values for premenopausal participants and triangles (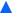) for postmenopausal. Values are mean ± SE. * Denotes that T3 is significantly different (*p* < 0.05) than T1.

**FIGURE 4:**
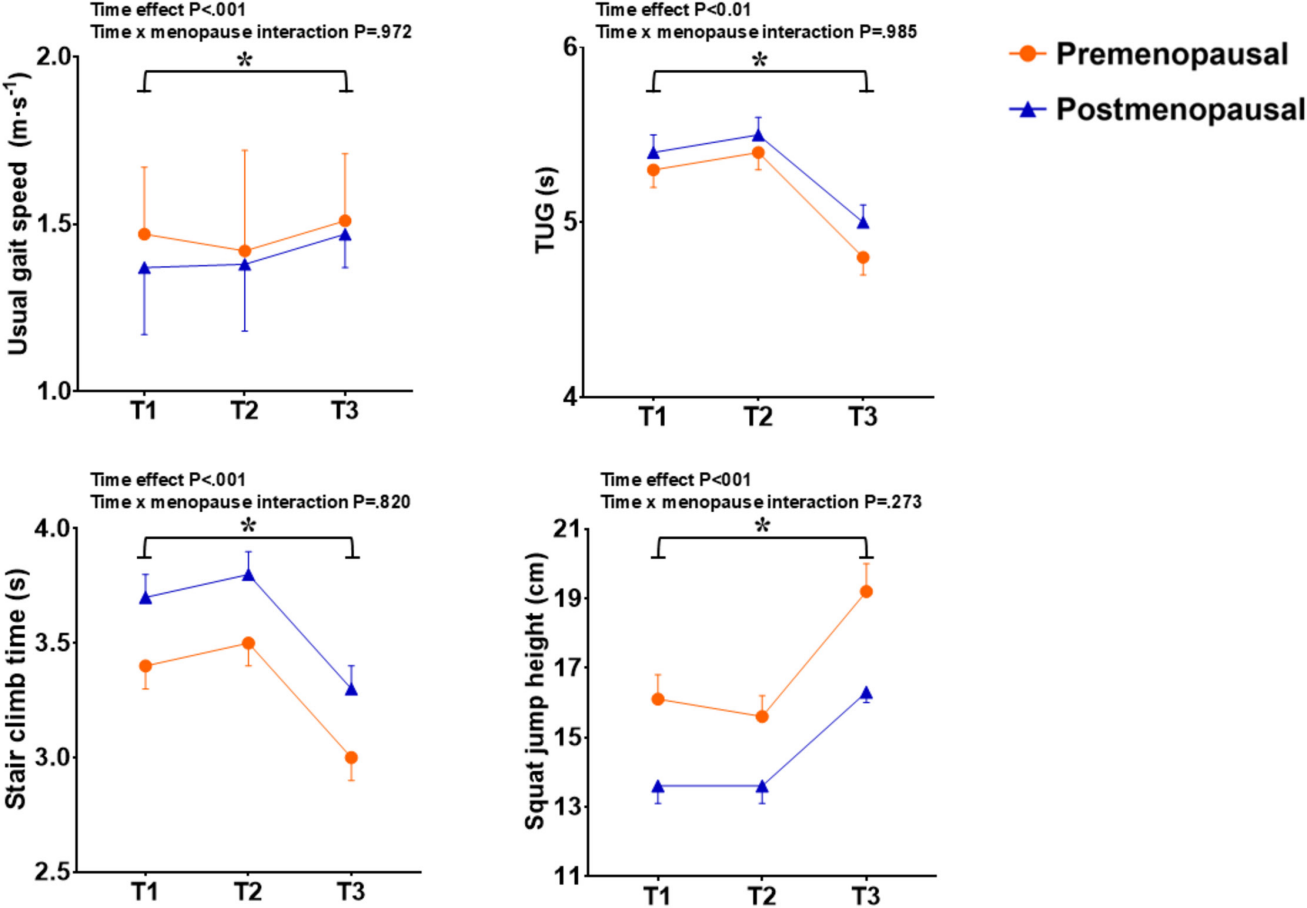
Physical function variables before (T1) and after (T2) 2 weeks of reduced physical activity and after a subsequent 12 weeks of exercise training (T3), according to menopausal status. (A) Usual gait speed; (B) timed up and go test; (C) stair climb time; and (D) squat jump height. Circles (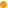) represent values for premenopausal participants and triangles (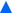) for postmenopausal. Values are mean ± SE. * Denotes that T3 is significantly different (*p* < 0.05) than T1.

Sixty‐seven volunteers completed the exercise training + nutrition phase and subsequent assessments (T3), from which 26 were premenopausal and 41 postmenopausal. Compliance rates for supplement consumption were 94.7% (FMP) and 90.0% (placebo). Sarcopenia‐related variables before and after the training period according to the supplementation group are shown in Table 3. All variables improved significantly after training (time effects *p* < 0.05), with no significant time × group interactions (*p* > 0.05). No time × menopausal status or time × age interaction was detected for any trait. Figures 2, 3, 4 display the sarcopenia‐related outcomes across timepoints according to menopausal status. Additional analyses revealed that sarcopenia traits were significantly increased compared to baseline (T3 vs. T1; *p* < 0.05), although HGS recovered only to baseline. During the exercise + supplementation phase, average energy intake was higher than at baseline (7554.8 vs. 8435.7 kJ, *p* < 0.05) due to higher carbohydrate (206.4 vs. 174 g/day; *p* < 0.05) and protein (94.9 vs. 82.9 g/day; *p* < 0.05) consumptions, but these differences did not persist when FMP or placebo were subtracted from the records. No differences were observed between FMP and placebo groups for total energy or macronutrient intake at any timepoint, except for a higher protein intake in the FMP group (101.4 vs. 89.8 g/day, *p* = 0.017), which disappeared when FMP or placebo nutritional values were subtracted from the analyses (i.e., higher intake resulted from supplementation being taken in addition to the normal diet).

**TABLE 3:** Mean and standard deviation values for muscle mass, muscle strength and physical function variables before (T2) and after (T3) the training period, according to study group.

Variables	Fortified milk powder			Energy‐matched placebo			Time effect	Time × group interaction
	T2	T3	∆%	T2	T3	∆%		
*N*	32			35				
Muscle mass variables								
LBM (kg)	42.6 ± 5.5	43.3 ± 5.2	1.8	44.3 ± 6.0	45.2 ± 6.0	2.3	< 0.01	0.42
LBM/body mass (%)	59.5 ± 5.3	60.6 ± 5.9	1.8	60.2 ± 4.9	61.1 ± 4.8	1.5	< 0.01	0.58
Appendicular LBM (kg)	18.2 ± 2.7	18.9 ± 2.7	3.8	18.7 ± 4.8	19.4 ± 4.8	3.7	< 0.01	0.94
Appendicular LBM/height^2^ (kg/m^2^)	6.8 ± 1.0	7.0 ± 0.9	2.9	6.9 ± 0.8	7.1 ± 0.8	2.9	< 0.01	0.94
Thigh muscle CSA (cm^2^)^a^	99.0 ± 20.0	102.4 ± 17.4	3.4	103.1 ± 14.1	108.4 ± 17.0	5.1	< 0.01	0.16
Thigh muscle density (HU)^a^	76.5 ± 1.6	77.0 ± 2.2	0.7	76.5 ± 2.2	77.3 ± 1.5	1.0	0.04	0.89
Shank muscle CSA (cm^2^)^b^	63.6 ± 9.7	66.5 ± 9.9	4.5	67.6 ± 8.2	69.8 ± 8.7	3.1	< 0.01	0.39
Shank muscle density (HU)^b^	77.6 ± 1.7	77.9 ± 1.6	0.4	76.9 ± 1.7	77.6 ± 1.8	0.9	< 0.01	0.28
Muscle strength variables								
HGS (kg)	23.3 ± 5.0	24.7 ± 5.6	4.8	24.4 ± 5.6	25.1 ± 6.3	1.3	0.02	0.46
HGS/body mass	0.33 ± 0.1	0.35 ± 0.1	5.5	0.33 ± 3.1	0.34 ± 0.1	1.4	0.07	0.29
Knee extensor PT (Nm)	121.4 ± 43.1	145.5 ± 37.5	18.8	120.6 ± 27.2	142.7 ± 35.1	18.3	< 0.01	0.70
Knee extensor PT/body mass (Nm/kg)	1.7 ± 0.5	2.0 ± 0.4	19.7	1.7 ± 0.5	2.0 ± 0.5	17.5	< 0.01	0.54
Plantar flexor PT (Nm)	86.9 ± 37.5	108.9 ± 35.5	25.2	81.4 ± 24.5	102.4 ± 28.7	25.8	< 0.01	0.86
Plantar flexor PT/body mass (Nm/kg)	1.2 ± 0.5	1.5 ± 0.5	25.1	1.1 ± 0.4	1.4 ± 0.4	23.2	< 0.01	0.65
Physical function variables								
Usual gait speed (m/s)	1.3 ± 0.2	1.5 ± 0.2	8.9	1.4 ± 0.3	1.5 ± 0.1	7.0	< 0.01	0.31
Squat jump height (cm)	14.3 ± 3.8	17.6 ± 4.5	22.5	14.3 ± 3.1	17.3 ± 3.8	21.0	< 0.01	0.64
Stair ascending time (s)	3.7 ± 0.7	3.2 ± 0.4	−13.5	3.7 ± 0.5	3.3 ± 0.4	−10.8	< 0.01	0.62
Stair ascending power (W)	350.2 ± 79.7	396.2 ± 73.7	12.5	347.8 ± 56.3	391.2 ± 53.7	12.5	< 0.01	0.76
TUG (s)	5.4 ± 0.7	4.8 ± 0.6	−14.3	5.5 ± 0.6	5.0 ± 0.5	−11.4	< 0.01	0.22

*Note:* a, *N* = 36; b, *N* = 46.

Abbreviations: CSA, cross‐sectional area; HGS, handgrip strength; LBM, lean body mass; PT, peak torque; TUG, timed up and go test.

Regarding adverse events, four participants reported muscle or joint pain during the exercise + nutrition phase. None required first aid or medical attention at the time of reporting. Two adverse events were reported for FMP. One reported stomach cramps, nausea, headaches and blood in the stool, and the other reported an initial laxative effect before also suffering from nausea and blood in the stool. Both were required to immediately cease FMP ingestion and returned to normal, with no further complications or need for treatment. They were recorded as lost to follow‐up (see Figure 1) and excluded from T3 data analyses.

## 4 Discussion

Sarcopenia is a progressive condition marked by reduced muscle mass, strength and function and related to adverse clinical outcomes. Menopausal transition seems to accelerate age‐related muscular decline and increase sarcopenia risk [15, 17]. In the present study, we examined changes in a variety of sarcopenia traits induced by 2 weeks of step reduction and a subsequent 12‐week period of exercise training with or without fortified milk powder supplementation in middle‐aged females. The prominent results indicated that, compared to baseline (T1), sarcopenia‐related variables were lower after 2 weeks of reduced physical activity, with statistical significance being noted for several muscle mass, strength and physical function measures (Table 2). Nonetheless, these variables not only fully recovered after the exercise programme but also exceeded baseline (T1) values, with no further benefits provided by the supplementation. We also sought to verify the interaction between menopausal status and these changes over time. As expected, menopausal participants were older and had significantly lower values for several sarcopenia variables at baseline, some of which persisted even after adjustment for age (Figure 2). Menopausal status, however, did not influence the changes provoked by step count reduction or subsequent exercise training. Taken together, these results corroborate the concept that a relatively short period of activity restriction contributes to skeletal muscle health reduction in healthy ageing females and that exercise involving resistance training, regardless of the increased protein supplementation (at least as provided in the present study), is an effective recovery strategy. The observations also suggest that, independent of age, menopause negatively impacts sarcopenia‐related variables but does not appear to impair the ability to adapt to exercise training.

Episodic periods of physical inactivity can occur as a consequence of illness, injury, poor weather conditions, hospitalization, carer responsibility or other phenomena, e.g., including the recent COVID‐19 pandemic, and might accelerate skeletal muscle atrophy and strength decline. The potential for acute periods of physical inactivity to accelerate age‐related losses of muscle mass and function has been labelled a ‘catabolic crisis’ [29] and has usually been investigated in individuals aged 60 years and older. For example, in healthy older adults, Breen et al. [22] showed that 14 days of reduced steps induced small but detectable LBM decreases, while one recent report [20] found that lean mass remained unchanged after 2 weeks of reduced steps in 70–80‐year‐old male and female subjects. In middle‐aged females, we observed limited loss of skeletal muscle size, with only shank CSA presenting a statistically significant decline. It is possible that the previously demonstrated downregulation of myofibrillar protein synthesis following 2 weeks of step reduction [22] does not translate into measurable changes in muscle volume or mass in the short term. The finding that the shank muscle was the only morphological variable to show a significant decline may be related to the pivotal role of the plantar flexors in gait. As the primary generators of propulsive force during walking, these muscles may be particularly sensitive to abrupt reductions in daily steps, potentially exhibiting signs of atrophy earlier than other muscle groups. Conversely, Reidy et al. [19] reported lean mass and knee extensor strength decreases after a similar period of reduced daily steps. The present study, however, is the first to explore the effects of step reduction in a wide range of sarcopenia‐related measures in middle‐aged females and to examine its interaction with menopausal status. Our findings indicate that 2 weeks of reduced physical activity are sufficient to impair several (but not all) measures of muscle mass, strength and function without an additional effect of menopausal status. These observations support the concept that menopause is associated with a poorer sarcopenia status but does not seem to affect their loss and recovery with activity restriction and exercise training, at least in middle age. It is important to note that this evidence is based on short‐term interventions, and the effects of prolonged or repeated reductions in physical activity or exercise training remain unclear.

There is clear evidence that exercise programmes including resistance training are effective strategies for the mitigation of muscle mass, strength and physical function losses induced by physical inactivity [20, 23, 29]. In a previous report, 4 weeks of exercise rehabilitation immediately after 2 weeks of step reduction in older adults not only promoted recovery of muscle strength and function but also provoked improvements above the baseline, preinactivity levels [20]. In the present study, similar results were found in middle‐aged females without the additional effect of menopausal status. Furthermore, we observed modest but significant LBM increases as a result of training, in contrast to Walker et al. [20] who reported no significant main effects for LBM in 70–80‐year‐olds. These differences might be attributed to a shorter training duration (4 weeks vs. 12 weeks in the present study) or to participants' ages (older vs. middle‐aged). Previous reports have shown that aged individuals present attenuated muscle volume enhancement [21] and myofibrillar protein synthesis rates [30] in response to retraining after inactivity compared to younger individuals. Although the postmenopausal females in the present study showed a preserved response to training stimuli, their baseline muscular traits were lower. Therefore, interventions to improve muscle strength and physical function should be emphasized in middle‐aged females, particularly after a period of physical activity restriction.

Addition of a fortified milk supplement had no additional effect above the exercise training + isoenergetic placebo (rice powder + whole milk powder) supplement in the present study. According to the diet records, participants followed instructions by taking the supplement in addition to their usual diet, since there was no difference between the groups when its content was removed, yet the supplement group had higher protein and CHO intake when the supplement was included in the analysis. Our sample comprised healthy 40–60‐year‐old females with adequate baseline nutritional intake, which may explain the lack of significant supplementation effect. Of course, individuals may underreport their dietary intake or reduce their intake during the recording period [31], although this is also likely to be consistent between the two groups. At baseline, daily protein intake was 1.1 g/kg/day, which is above the recommended minimal RDI of 0.8 g/kg/day. Future studies are needed to clarify the potential additive effects of dairy supplementation to exercise training, particularly in more frail sections of the population or using a supplement with different ingredient mixes (e.g., higher protein content). Exercise training with or without nutritional supplementation is key to improving muscular health in different populations [32, 33, 34], including in middle‐aged females [15, 18, 35]. However, nutritional strategies using proteins, food fortification and supplementation can amplify the effects of exercise on muscle mass and strength, with potential to accelerate recovery after a period of disuse [25, 29, 36, 37]. Using a formula identical to ours, also in middle‐aged females, Daly et al. [38] found that the supplement did not enhance the effects of a 4‐month resistance‐based training programme on muscle strength and performances in a number of physical function tests but did promote greater improvements in body composition, standing balance, aerobic fitness or bone metabolism. Of note, the study [38] did not impose a previous period of reduced physical inactivity, participants were slightly older, less active and with higher BMI at study commencement, the training duration was longer (4 months vs. 12 weeks), and a greater emphasis was placed on resistance training exercises. Nonetheless, these data indicate a small positive effect of additional supplementation, which might promote greater longer term adaptations. Positive effects of dairy product ingestion additional to resistance training on muscle protein synthesis have been reported in young male individuals [25, 26] and a systematic review reported that nutrient‐rich dairy products may improve sarcopenia traits in older people [37]. However, Kukuljan et al. [27] found that the ingestion of 400 mL/day of low‐fat fortified milk (836 kJ, 1000‐mg calcium, 800 IU vitamin D and 13.2 g of protein) did not amplify the effects of resistance training over an 18‐month period on muscle mass, strength or physical function in healthy middle‐aged to older men (50–79 years). The present double‐blind study is the first to investigate the effects of fortified milk with 18 g of protein in conjunction with exercise training on the recovery of muscular health after a reduced physical activity period in middle‐aged females. In this case, an effect could not be detected above that already provided by the exercise training alone.

The present study contains both strengths and limitations. That no previous studies had examined the effects of reduced physical activity and subsequent retraining on sarcopenia traits in middle‐aged females is a novel aspect. Additionally, we explored the interaction of menopausal status on the observed changes and whether the addition of a fortified milk supplement might enhance exercise‐induced adaptations. A comprehensive set of sarcopenia‐related traits was included, which were assessed using gold standard procedures and a double‐blind approach to evaluate the effects of the supplement against an isocaloric placebo. Because of the scope of the study, it was not possible to additionally include nonreduced step and nonexercise control groups, which is a limitation of the study. However, the significant losses and recoveries of numerous variables over time (i.e., as opposed to a general progressing over the study period) are suggestive that the interventions themselves were the source of effects. Additionally, the timing of supplement intake was recommended, but not controlled. Previous evidence [39] suggests that the timing of postexercise protein intake may influence muscle hypertrophy, although a recent systematic review [40] indicates that the beneficial effects of supplementation on LBM are independent of timing. Baseline circulating vitamin D levels were not assessed, which may have influenced the findings given the increasingly recognized role of vitamin D for sarcopenia‐related traits [41]. Finally, this study was conducted in middle‐aged females, and thus, the data should not be considered reflective of the changes that might be observed in males or participants of other ages.

The present results show that 2‐week step count reduction in middle‐aged females is sufficient to negatively affect muscle strength and physical function, as well as muscle size to a lesser degree, which are traditional markers of sarcopenia. However, menopausal status did not interact with these reductions despite postmenopausal females having statistically poorer baseline values, placing them closer to sarcopenia thresholds. The broad‐based exercise training programme, including strength exercises, not only recovered muscular health but also promoted improvements above baseline, indicating that exercise may be a powerful intervention to reduce sarcopenic traits after periods of inactivity, even in middle‐aged females. The addition of a FMP in addition to the normal diet during the training period did not enhance exercise‐induced muscular adaptations in the current, healthy cohort, when compared with a rice + dairy milk placebo, although it may be that other supplement formulations could be found to be effective in future investigations, including in individuals with poorer health status.

## Conflicts of Interest

S.D.P. held the Fonterra Chair in Human Nutrition at the University of Auckland, New Zealand.

## Supporting information




**Data S1:** Supporting information.
