# Supplementary material for: Postinactivity Exercise Training Improves Sarcopenia Traits in 40–60‐Year‐Old Women Regardless of Fortified Milk Supplementation
Source: J Cachexia Sarcopenia Muscle. 2025 Nov 3;16(6):e70080. doi: 10.1002/jcsm.70080 (PMC12582911; doi:10.1002/jcsm.70080)
Supplement: Supplementary file 1 — Data S1: Supporting information. [file JCSM-16-e70080-s001.docx]

**Supplementary File: Methods**

**Exclusion criteria**

Exclusion criteria included: 1) aged <40 or >60 y; 2) BMI < 18 or > 35 kg/m^2^; 3) being engaged in regular exercise training; 4) previous negative reaction to milk or dairy proteins; 5) previous negative reaction to fish; 6) currently perform structured physical activity in their occupation; 7) alcohol > 4 drinks per week; 8) smoking (or only having stopped smoking within the last 2 years); 9) having any fixations (i.e. spine or femoral rods, hip replacements); 10) present but irregular menstrual cycle (i.e. not within 20-35 days); 11) using hormone replacement therapy; 12) planning to become pregnant or currently breastfeeding; 13) using supplements such as vitamin D (> 400 IU), calcium, bone/joint assistants (glucosamine, chondroitin), or antacids containing calcium carbonate, and not willing to stop for at least 6 weeks prior to, or throughout, the duration of the trial; 14) anti-inflammatory use (including aspirin, ibuprofen, omega 15); Use of beta blockers or ACE inhibitors; 16) metabolic bone disease (including osteoporosis, osteomalacia, osteogenesis imperfect and Paget’s disease); 17) endocrine disorders (including hyper- or hypo-thyroidism, parathyroid disease, diabetes mellitus and Cushing’s syndrome); 18) cancer (including cervical, ovarian, uterine, breast, liver and of the gastrointestinal system); 19) other medical conditions impacting on digestion, including diseases of the gastrointestinal tract, or pervious operations such as gastrectomy or intestinal resection, or the liver (i.e. cirrhosis); 20) any chronic muscle disease, disorder or injury; 21) asthma that is not controlled or other respiratory diseases; 22) self-reported personal or family history of deep vein thrombosis or similar clotting disorders.

Those wishing to continue then attended a screening session that included dual-energy X-ray absorptiometry (DEXA) (Horizon A, Hologic, Washington, USA) of the hip and lumbar spine and completed a food frequency. Participants were further excluded if bone mineral density T-score was < -2.5 at either region; protein intake was > 1.2 g/kg/day; or calcium intake was > 750 mg/day.

**Block randomisation**

Following confirmation of eligibility, all participants were given a unique sequentially assigned participant number, which had previously been randomised into one of two treatment arms (supplement-plus-exercise or placebo-plus-exercise) using block randomised structure (blocks of 10 participants). Participants and research staff at the study centres were blinded to treatment allocation. Research staff prepared the treatments as instructed by the manufacturer and gave to the participants in identical sachets labelled according to regulatory requirements and marked with the participant number. The randomisation lists and participant identifiers will be confidential to the Fonterra Co-operative Group Ltd representative.

**Muscle strength evaluation**

Handgrip strength (HGS) was measured bilaterally (Jamar hydraulic dynamometer; Sammons Preston Rolyan, IL, USA). All measurements were performed in a standing position with shoulder joint adducted and neutrally rotated, elbow joint slightly bent, forearm in the neutral position, and wrist between 0° and 30° of extension on self-selected basis. Handle position for the majority of participants was position two, but this was altered for those who need a smaller or larger grip distance. Each participant performed three trials with each arm with 30 s between each measurement in order to minimize muscle fatigue. The mean between the highest value of each arm in kilograms was used for analysis.

Knee extensor and plantar flexor isometric torques were evaluated using an isokinetic dynamometer (Biodex 4, Biodex Medical, Inc., Shirley, NY, USA). Participants performed a 5-min warm up on a cycle ergometer with light load (60 rpm, 1 kp; Monark cycle ergometer, Monark, Sweden). Participants were then seated in the isokinetic dynamometer seat so that the lateral condyle of the knee or lateral malleolus of the ankle joint lined up with the axis of the dynamometer’s lever arm. Following a detailed reminder of the procedures, participants performed 3-s isometric knee extensor contractions at 50, 70 and 90% of their perceived maximum at 90^o^ knee angles using their right leg as further warm-up and familiarization. Then they performed three maximal 5-s knee extensions with 30 s of passive rest between. Participants were instructed to perform the test “as fast and then as hard as possible” and verbal encouragement was provided. After completing the knee extensor tests, the participants plantar flexor torque was tested with the ankle in the neutral position (0°). They performed three maximal 3-s plantar flexions separated by 30 s of passive rest with the same instruction and encouragement as for the knee extensor test. Trials at either joint showing a drop in joint torque > 5 Nm at the start of contraction were discarded and another trial completed. The highest reading at each joint was recorded for analysis. Equipment calibration was performed according to the manufacturer’s specifications before each testing session. Torque values were analyzed in absolute values (Nm) and relative to body mass (Nm/kg).

**Physical function tests**

The timed up and go (TUG), participants were seated in a standard chair with their backs straight, feet shoulder apart and flat on the ground. A trained assessor recorded the time taken for participants to, on the “Go” signal, rise from the chair, walk 3 m as fast as possible but without running, turn around a cone and sit back on the chair. Two attempts with 30-s rest intervals were performed and the fastest time was recorded for analysis.

For the ‘usual gait speed’ 6-MWT, participants were instructed to walk “at your usual walking speed, just as if you were walking down the street to go to the shops” across a 10-m flat walkway. Markers were used at 2 and 8 m, and the time to walk 6 m was recorded using a stopwatch when the participants trunk (identified by the hip joint) crossed the start and finish lines. Time was recorded to the nearest 0.01 s, the test was repeated after 30 s of rest, and the mean result was taken for analysis. Gait speed was then calculated.

Squat jump height was evaluated as an indicator of ‘muscle power’. Participants first completed trials at 50, 70 and 90% of perceived maximum exertion as warm-up/familiarization. The test was then initiated with participants standing with both feet without shoes in the center of a force platform (Kistler Instrumente, Switzerland) approximately shoulder width apart and with the hands placed on the hips. Once the force platform was triggered for data collection, the participants squatted to a self-selected depth, held for 1 s, jumped “as high as possible”, then landed on both feet on the platform. Three attempts were performed with 30-s rest intervals. Jump heights were calculated and the best trial recorded. Trials were repeated when a countermovement was visually observed. Finally, the time required to ascend a flight of 11 stairs each 16 cm high, without missing a step, was evaluated. Participants started at the base of the stairs with both feet together and then ascended the stairs on the ‘go’ command of the experimenter. Timing commenced when the participant made their first movement and stopped when both feet were placed on the final step. Participants had one familiarization trial, after which two trials were recorded to the nearest 0.1 s. One-min of rest was provided between the ascents. The fastest stair ascent time was taken as stair climb performance, and stair climb power was also measured using the equation: Power (watts) = 9.81 × body mass (kg) × vertical height (m) / time (s).

**Peripheral Quantitative Computed Tomography**

Lower leg and thigh whole muscle cross-sectional areas were determined using peripheral quantitative computed tomography scans (pqCT; XCT 3000; Stratec Medizintechnik, Pforzheim, Germany). Thigh muscle cross-sectional area (CSA) was measured at the midpoint between the lateral epicondyle and greater trochanter with participant seated on a height-adjustable chair, right leg in the bore of the scanner, and the left leg supported outside the bore. Bore position was carefully adjusted so that the scan was completed at the targeted site. Lower leg CSA was subsequently obtained using the same protocol, with the participant’s position adjusted to scan at shank levels of 4% and 66% from the distal end of the tibia, as located by a scout scan. From the mid-thigh and 66% lower leg scans, intramuscular and subcutaneous tissues were separated from the muscular area to allow determination of CSA and muscle density using the integrated Stratec software using a median filter mode for noise reduction (F03F05). Due to equipment technical difficulties during the study, only 36 and 46 participants successfully completed pqCT evaluations for thigh and shank, respectively, at all timepoints.

**Body composition assessment**

Body mass and height were measured using standard procedures and body mass index (BMI) was calculated. Body composition was measured at all timepoints using dual-energy X-ray absorptiometry (DXA) (Horizon A, Hologic, Washington, USA). For the procedure, volunteers laid supine on the DXA scanner table with body carefully centered. The software (Hologic) provided fat mass, lean body mass (LBM) and bone mineral content for the whole body as well as for the arms, legs, and trunk separately. Appendicular LBM was calculated as the sum of both arms and legs. LBM was also calculated relative to body mass (%) while appendicular LBM was also considered relative to height squared (kg/m^2^). Percent body fat was used to characterize each participant’s adiposity. The equipment was calibrated daily according to manufacturer specifications and all procedures were conducted by the same trained professional.

**Supplementation**

Participants were randomly assigned to the fortified milk supplementation or to a placebo group. The fortified milk powder was ingested in two 30-g doses per day mixed with 150 ml water. The supplement was largely comprised milk powder (skim and whole), and contained 9 g protein, 16.5 g carbohydrate, 0.7 g fat, 634 mg calcium, 7.5 µg Vitamin D, 86 mg magnesium, and 2 mg zinc per serve. It also contained low levels of vitamins B12, B6, E and C and a small amount of hydrolyzed fish collagen. The placebo powder was also ingested in doses of 30 g twice a day and was energy matched to the supplement powder (442.9 kJ vs. 486.4 kJ, respectively), and predominantly contained carbohydrates (rice powder and whole milk powder), along with 1.6 g protein, 25 g carbohydrate, 1.6 g fat, 29 mg calcium, and no added vitamins or minerals. The supplement was designed to be additional to the meal, not a replacement, and the participants were asked to consume these servings with breakfast and lunch on non-exercise days, and to consume one of dose within 30 min of exercise session completion on their exercise days.

Participants received their supplement sachets in four instalments with a new batch every 2.5 weeks and were asked to take the supplement in addition to, not instead of, their normal food intake. The first group of sachets (along with ingestion guidelines) were dispensed to the participants at the first testing session. To ensure supplementation compliance, the participants were required to return all used and unused sachets every 3 weeks. A log of the sachets dispensed (out) and sachets returned (in) was kept for each participant and the balance calculated. Supplements and placebo were implemented in a double-blind fashion. The first author (JT) was responsible for participant recruitment and coordination of training and testing procedures. However, participant allocation (within blocks of 10) was performed by the principal investigator (AB), who did not participate in assessments or training. The allocation sequence was concealed from the first author, the assessment team, and the participants to ensure double-blind conditions.

**Diet records**

Three-day reported weighed dietary records were obtained using a booklet with pages for recording each day's food intake at T1; during the final 3 days of reduced activity, pre T2; and during the 11 week of training, pre T3. Scales and measuring cups/spoons were provided. Before the recording, participants received full verbal instructions for the diary completion and then retained a written instruction booklet, and examples were also provided. All food and beverage details were recorded, including a description of the foods, brand names, and the mass of the food where possible or measures of the amount consumed (in units of grams, cups, teaspoons, pieces, slices, etc.). Household weights and measures were subsequently converted to food masses and the data were then analysed using the Foodworks© 8 software (Xyris Software, Australia). After data entry, each record was verified by a second researcher and corrections made if errors in data entry were observed.

**Sample size**

Research papers examining changes in strength and power performance, for example using the stair climb power test, were examined. Data comparing performance changes within groups as well as between-group comparisons showing differences in responses when dietary (e.g. protein supplementation) or exercise (e.g. heavy vs. fast strength training) interventions were imposed between groups, were gathered. Studies involving bed rest or other detraining interventions were also examined. Effect sizes were calculated as the change in performances, or differences in the changes between groups, relative to the pre-intervention standard deviation (of the control group if it was clearly specified). These effect sizes were commonly large and ranged 1.2 – 1.6.

We began by using a more conservative effect size of 1.0 because our participants would be younger, the supplement would be lower in protein (which may speculatively produce less effect on stair climb time), and the training would be less focussed on heavy strength training (which may speculatively produce less effect). In this case, the mean difference in the changes between two groups was equal to the average variability between subjects before the intervention. We assumed a single outcome variable could be compared using t-test procedures.

The outputs indicated that a sample size of 46 would give a power of 0.9 for a conservative effect of 1.0 (i.e. smaller than the 1.2 – 1.6 magnitudes found between groups in studies in older individuals, using either heavy resistance training, or using higher protein supplementation). For an even more conservative effect size of 0.8, a sample size of 68 was obtained for a power of 0.9.

**Adverse events**

Participants were required to report any exercise or supplement related adverse events. The exercise session trainers were also required to report any adverse events that participants mentioned to them during the sessions. Exercise adverse events related to any event requiring a modification or removal of an exercise, or any movement difficulties following the exercise session thought to be caused by the exercise. Supplement related adverse events related to any adverse physical responses to the supplements (i.e. digestive problems).
